# Conjunctival ultraviolet autofluorescence as a biomarker of outdoor exposure in myopia: a systematic review and meta-analysis

**DOI:** 10.1038/s41598-024-51417-9

**Published:** 2024-01-11

**Authors:** Natali Gutierrez Rodriguez, Aura Ortega Claici, Jorge A. Ramos-Castaneda, Jorge González-Zamora, Valentina Bilbao-Malavé, Miriam de la Puente, Patricia Fernandez-Robredo, Sandra Johanna Garzón-Parra, Manuel Garza-Leon, Sergio Recalde

**Affiliations:** 1 Grupo de Investigación en Optometría-Facultad de Optometría de la Universidad Antonio Nariño, Bogotá, Colombia; 2 Retinal Pathologies and New Therapies Group, Experimental Ophthalmology Laboratory, Department of Ophthalmology, Clinica Universidad de Navarra, Pamplona, Spain; 3 Faculty of Medicine, Universidad de Navarra, Pamplona, Spain; 4 Research Group Innovación y Cuidado, Faculty of Nursing, Universidad Antonio Nariño, Neiva, Colombia; 5 Department of Ophthalmology, Clínica Universidad de Navarra, Madrid, Spain; 6 Navarra Institute for Health Research, IdiSNA, Pamplona, Spain; 7 Department of Ophthalmology, Bellvitge University Hospital, Barcelona, Spain; 8 Clinical Science Department, Science of Health Division, University of Monterrey, San Pedro Garza García, Nuevo León México

**Keywords:** Biomarkers, Medical research

## Abstract

Outdoor exposure is considered the primary modifiable risk factor in preventing the development of myopia. This effect is thought to be attributed to the light-induced synthesis and release of dopamine in the retina. However, until recent years, there was no objective quantifiable method available to measure the association between time spent outdoors and myopia. It is only recently that the conjunctival ultraviolet autofluorescence (CUVAF) area, serving as a biomarker for sun exposure, has begun to be utilized in numerous studies. To provide a comprehensive summary of the relevant evidence pertaining to the association between the CUVAF area and myopia across different geographic regions and age groups, a systematic review and meta-analysis were conducted. The search encompassed multiple databases, including MEDLINE, SCIENCE DIRECT, GOOGLE SCHOLAR, WEB OF SCIENCE, and SCOPUS, and utilized specific search terms such as "conjunctival ultraviolet autofluorescence", "CUVAF", "UVAF", "objective marker of ocular sun exposure", "myopia", "degenerative myopia", and "high myopia". The bibliographic research included papers published between the years 2006 and 2022. A total of 4051 records were initially identified, and after duplicates were removed, 49 articles underwent full-text review. Nine articles were included in the systematic review. These studies covered myopia and outdoor exposure across different regions (Australia, Europe and India) with a total population of 3615 individuals. They found that myopes generally had smaller CUVAF areas compared to non-myopes. The meta-analysis confirmed this, revealing statistically smaller CUVAF areas in myopic patients, with a mean difference of − 3.30 mm^2^ (95% CI − 5.53; − 1.06). Additionally, some studies showed a positive correlation between more outdoor exposure and larger CUVAF areas. In terms of outdoor exposure time, myopic patients reported less time outdoors than non-myopic individuals, with a mean difference of − 3.38 h/week (95% CI − 4.66; − 2.09). Overall, these findings highlight the connection between outdoor exposure, CUVAF area and myopia, with regional variations playing a significant role. The results of this meta-analysis validate CUVAF as a quantitative method to objectively measure outdoor exposure in relation with myopia development.

## Introduction

Myopia is the most prevalent refractive error worldwide and it is estimated that its progression will mean that by 2050, half of the world's population will be myopic^1,2^. It also represents an important public health problem, since it can progress to become a cause of irreversible visual disability^3^, due to pathologies such as glaucoma, cataract, retinal detachment and myopic maculopathy^4^. For the last two decades, the increase in prevalence in Asia has been very dramatic, where it already exceeds 52% of the population. However, this trend is not only seen in Asian countries, but also in both Europe and the USA, where the prevalence of myopia is above 30%^5^.

The multifactorial origin of myopia could be attributable to both genetic and environmental factors. Yet due to the current trend towards urbanization, it is believed that environmental factors, such as increased near work activities and decreased time spent outdoors, may play a major role in the recent increase in myopia worldwide^6–8^. Of these two factors, time spent outdoors has been the most studied, with several randomized clinical trials conducted worldwide to assess the effect of outdoor exposure on the prevention of myopia^9–16^. Although the exact mechanisms remain unclear, it is hypothesized that outdoor light stimulates dopamine synthesis in the retina which could mediate eye growth among other functions^17–20^.

**Figure 1:**
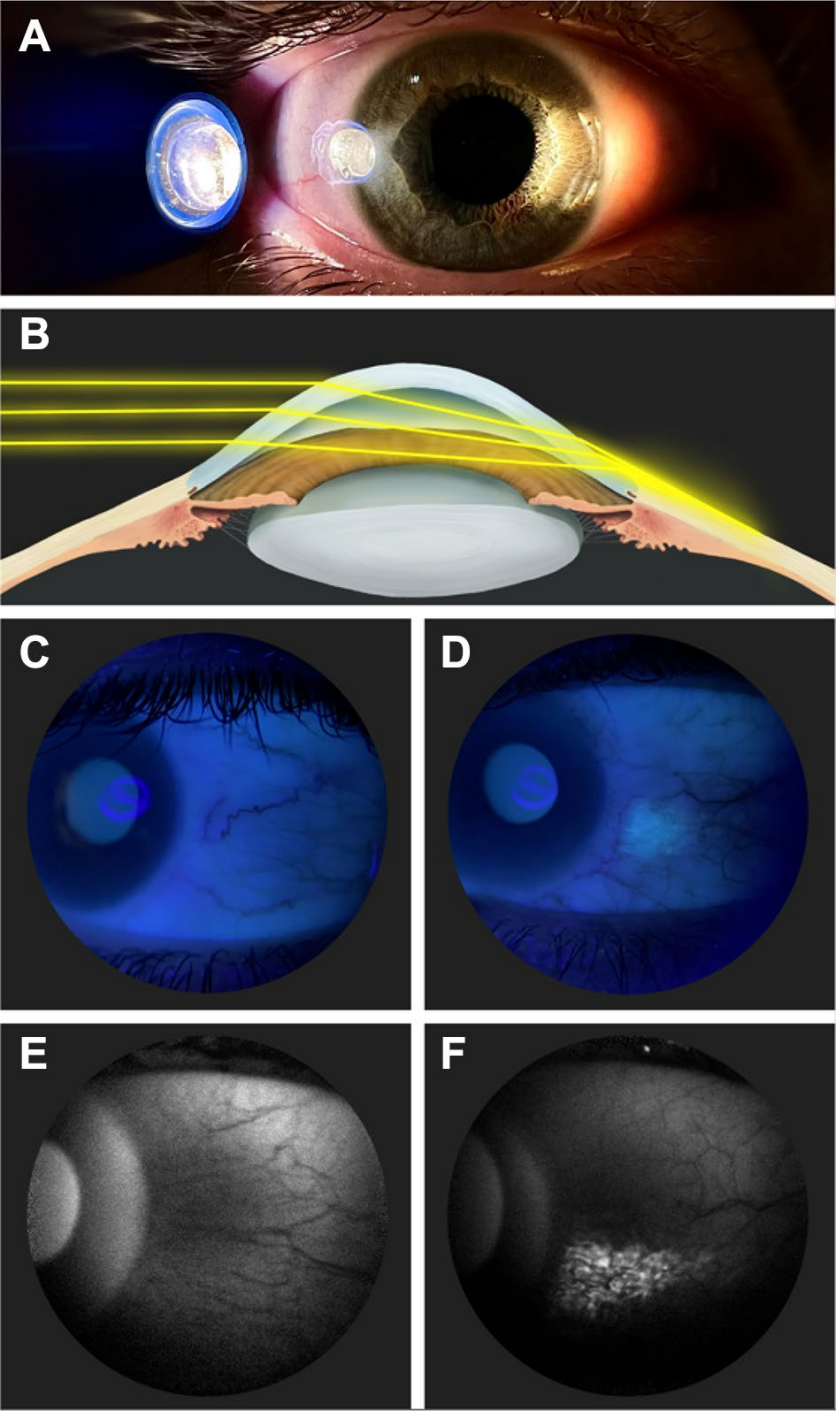
(**A**) In vivo demonstration of corneal focusing of peripheral light coming from the temporal side of the eye onto the limbus and nasal conjunctiva with higher light intensity compared to the temporal side. (**B**) Optical representation of the peripheral light focusing effect that leads to a concentration of the incoming rays of light, passing through the anterior chamber, onto the contralateral limbo-conjunctival area of the eye. (**C**) CUVAF negative (no conjunctival hyperautofluorescent area is seen) in a color photograph taken under UV light (peak wavelength of 365 nm). (**D**) CUVAF positive (demonstrates an area of hyperautofluorescence in the right nasal interpalpebral region) in a color photograph taken under UV light (peak wavelength of 365 nm). (**E**) CUVAF negative (no conjunctival hyperautofluorescent area is seen) in a photograph taken using the BAF mode of the Heidelberg Spectralis HRA + OCT (peak wavelength of 488 nm). (**F**) CUVAF positive (demonstrates a triangular conjunctival hyperautofluorescent area with limbar base and temporal apex) in a photograph taken using the BAF mode of the Heidelberg Spectralis HRA + OCT (peak wavelength of 488 nm).

While questionnaires have traditionally assessed outdoor time, they have limitations which include recall bias and inaccuracies^21,22^. Therefore, objective methods are being investigated. An emerging, non-invasive and quantifiable biomarker for outdoor time is conjunctival ultraviolet autofluorescence (CUVAF)^23–25^, CUVAF was first described as a localized area of autofluorescence under ultraviolet (UV) light in the bulbar conjunctiva^23^, probably due to the corneal focusing effect of peripheral light (Fig. 1), and is thought to represent preclinical lesions of UV-induced conjunctival damage^26–28^. To detect the area of CUVAF, a special custom-built device was designed. Subsequently, Lingham et al. validated an optical coherence tomography (OCT) device commonly used in clinical practice (Heidelberg Spectralis HRA + OCT) (Fig. 1)^29,30^. Although different devices were used, the technique for obtaining the CUVAF area is based on the same procedure.

Various studies have shown an inverse relationship between CUVAF and myopia^24,31–34^, making it a promising, cost-effective, and reproducible tool for routine eye care. Nevertheless, these studies encompassed diverse populations with variations in age and geographic location, which may affect CUVAF measurements. Hence, the present systematic review and meta-analysis aim to assess the link between CUVAF and myopia, while considering factors like outdoor exposure, geographical location, and participant age.

## Methodology

### Eligibility criteria

In this systematic review and meta-analysis: cohort, cross-sectional, and case–control studies were selected. The compiled literature comprised studies investigating the association between CUVAF area and myopia. Research papers published between the years 2006 and 2022 which used the CUVAF technique as a biomarker of outdoor activity in myopes were included. Letters to the editor, review articles, case reports, incomplete texts, theses, and articles written in a language other than English were excluded. Experimental animal studies or those using CUVAF parameters in pathologies other than myopia were also excluded. Duplicates were identified using the Covidence platform (Covidence systematic review software, Veritas Health Innovation, Melbourne, Australia) and confirmed manually.

### Search strategy

The search was performed in the following databases: MEDLINE, SCIENCE DIRECT, GOOGLE SCHOLAR, WEB OF SCIENCE and SCOPUS, using the following search terms: "conjunctival ultraviolet autofluorescence", "CUVAF", "UVAF", "objective marker of ocular sun exposure", "myopia", " degenerative myopia", "high myopia" and was performed between June and August 2022. Each primary article obtained from the search was screened for inclusion. To ensure inclusion of all relevant research papers, references of selected studies were manually reviewed.

### Selection process

**Figure 2:**
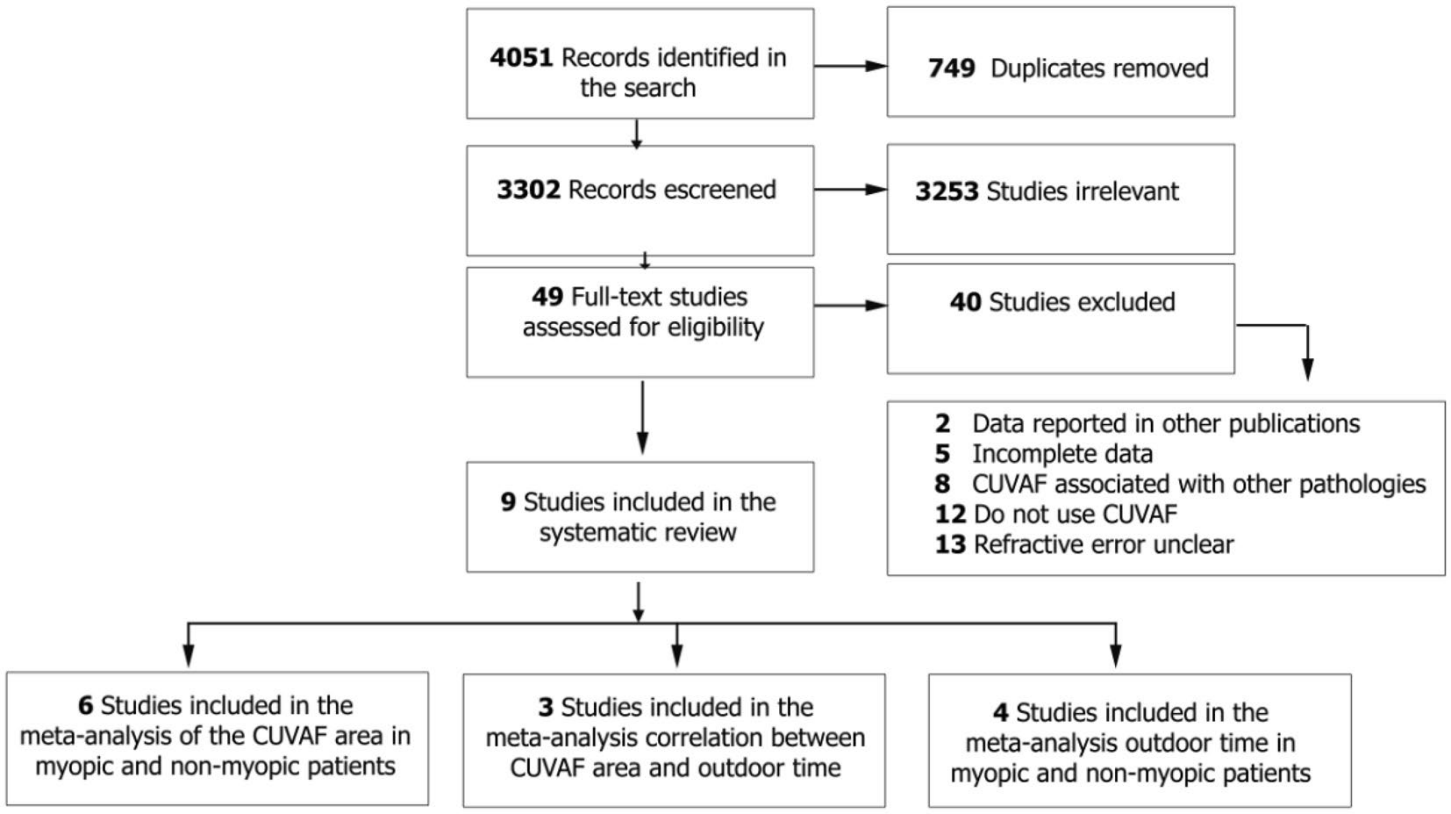
Flowchart of the literature search and study selections.

Articles were selected and analyzed by 5 independent authors according to the selection criteria using the Covidence tool. Titles and abstracts were reviewed by ASO, MAG and JNG; full-text review was performed by ASO and JNG assessing eligibility criteria. Discrepancies were resolved by SRM, MAG. The main reason for rejecting articles was based on incompleteness of the reported information (Fig. 2).

Data extraction was performed by JAR, SRM, ASO, JNG and MAG, analyzing the following variables: main author, year of publication, type of study, population, age, educational level, measurement equipment used, sex, axial length (AL), spherical equivalent (SE), CUVAF area and outdoor exposure time.

### Assessment of the methodological quality

Risk of bias was assessed using the Newcastle Ottawa Scale (NOS) for analytical observational studies^35,36^ which assesses the risk of bias during selection, comparability, and outcome. For analytical cross-sectional studies, the Joanna Briggs Institute instrument was used for evaluation of inclusion criteria, description of subjects, measure of exposure, measure of the condition, identifying confounding factors and strategies to deal with confounding factors, measure of outcome, and statistical analysis^37^.

### Synthesis of information

A qualitative analysis of each of the articles was performed taking into account the characteristics and type of the studies, the population included, the technique used to measure CUVAF area and the values of CUVAF area, AL, and average time spent outdoors.

### Quantitative analysis

A first meta-analysis was performed which assessed the difference in the mean CUVAF area of myopic compared to non-myopic individuals (n = 6). In a second meta-analysis, the mean difference between time spent outdoors in myopic patients compared to non-myopic patients was determined (n = 4). In a third meta-analysis, the overall result of the correlation coefficient of CUVAF area with time spent outdoors in myopic patients was calculated (n = 3). For the three meta-analyses, the mean difference with its 95% confidence interval was calculated.

A subgroup analysis was performed, according to the geographical location of the study (Australia-Asia and Europe) using a random effects model, and heterogeneity was assessed using the I2 statistic test. Finally, a meta-regression analysis was performed. All analyses were performed in RStudio software (version 2022.12.0) using the meta library.

## Results

### Systematic review

A total of 4051 records were identified in the databases. After removing duplicates, the titles and abstracts of 3302 records were reviewed and 3253 were excluded at this stage. 49 articles were subject to a full-text review, of which 9 articles were included in the systematic review^24,31–34,38–41^ (Fig. 2).

The characteristics of the included studies are summarized in Table 1. Of the total, six studies were conducted in Australia^24,32,38–41^, two in Europe^31,33^ and one in India^34^. The articles were published between 2012 and 2022 and included a total population of 3615 individuals. Outdoor exposure time was measured by means of self-reported questionnaires.

**Table 1:** Characteristics of the studies included in the systematic review and meta-analysis.

Main author (year)	Country	Type of study	Population	Number of participants	Age of participants (years)	Mean CUVAF area in myopes vs non-myopes (mm^2^)	Outdoor exposure time (OET) (h/week)
Sherwin et al. (2012)^24^	Australia	Cross-sectional	White	636	15–89	Median was lower in subjects with myopia (SE ≤ − 1.0 D), 16.6 mm^2^ vs. 28.6 mm^2^ in non-myopic patients; P: 0.001; and was also lower using the SE ≤ − 0.5 D, 24.5 mm^2^ vs. 28.6 mm^2^, P: 0.012	NA
Yazar et al. (2014)^32^	Australia	Cross-sectional	European white and Asian	946	18.3– 22.1	Presence of myopia was inversely associated with higher CUVAF area	NA
Mcknight et al. (2014)^41^*	Australia	Cross-sectional	European white and Asian	1328	20.1	Mean CUVAF area in myopic patients 37.3 ± 29.9 mm^2^ vs. CUVAF area in non-myopic patients 50.8 ± 34.3 mm^2^	NA
Kearney et al. (2018)^33^	UK	Cohort	European	54	18–20	Mean CUVAF area in myopic patients 4.2 ± 3.7 mm^2^ vs. CUVAF area in non-myopic patients 6.7 ± 6.1 mm^2^	NA
Charng et al. (2019)^40^*	Australia	Cross-sectional	European white and Asian	992	Gen 1: 40.8 Gen2: 26.6	Gen1: mean CUVAF area in myopic patients 27.72 ± 27.79 mm^2^ vs. CUVAF area in non-myopic patients 29.76 ± 26.36 mm^2^ Gen 2: mean CUVAF area in myopic patients 42.66 mm^2^ ± 29.37 vs. CUVAF area in non-myopic patients 46.79 ± 31.63 mm^2^	NA
Kumar et al. (2021)^34^	India	Case control	Indian	120	10–25	Mean CUVAF area in myopic patients 10.81 ± 15.14 mm^2^ vs. CUVAF area in Non-myopic patients 2.01 ± 3.05 mm^2^	Mean OET in myopic patients 3.1 ± 3.2 h/week vs. OET in non-myopic patients 6.3 ± 6.6 h/week
Lingham et al. (2021)^38^	Australia	Cohort	Caucasian	303	25.3–30	Mean CUVAF area in myopic patients 28.7 ± 23.7 mm^2^ vs. CUVAF area in non-myopic patients 37.2 ± 24.5 mm^2^	Mean OET in myopic patients 11.13 ± 1.18 h/week vs. OET in non-myopic patients 14.63 ± 1.69 h/week
Lee et al. (2022)^39^*	Australia	Cohort	European white and Asian	516	20–28	Mean CUVAF area in myopic patients 41.8 ± 29.3 mm^2^ vs. CUVAF area in non-myopic patients 41.1 ± 34 mm^2^	Mean OET in myopic patients 12.6 ± 12.4 h/week vs. OET in non-myopic patients 19.1 ± 21.1 h/week
Bilbao et al. (2022)^31^	Spain	Cross-sectional	European	228	22	Cohort 1: mean myopia 2.2 ± 1.9 mm^2^ vs. non-myopic 3.7 ± 4.4 mm^2^ Cohort 2: mean myopia 1.7 ± 1.8 mm^2^ vs. non-myopic 4.0 ± 2.6 mm^2^	Mean OET in myopic patients 7.8 ± 4.8 h/week vs. OET in non-myopic 9.3 ± 5.7 h/week

*The population of these studies is part of the study cohort in Australia (Raine)^25^.

Eight studies obtained the CUVAF measurement by custom-made photographic systems^24,32–34,38–41^ and one study using the Heidelberg OCT system^31^. All studies report that myopes have significantly smaller CUVAF areas than non-myopes.

The relationship between CUVAF area and outdoor exposure time (OET) was evaluated in four articles. A positive correlation between more time spent outdoors and larger CUVAF areas was reported in only two of the articles^38,41^.

### Meta-analysis

**Figure 3:**
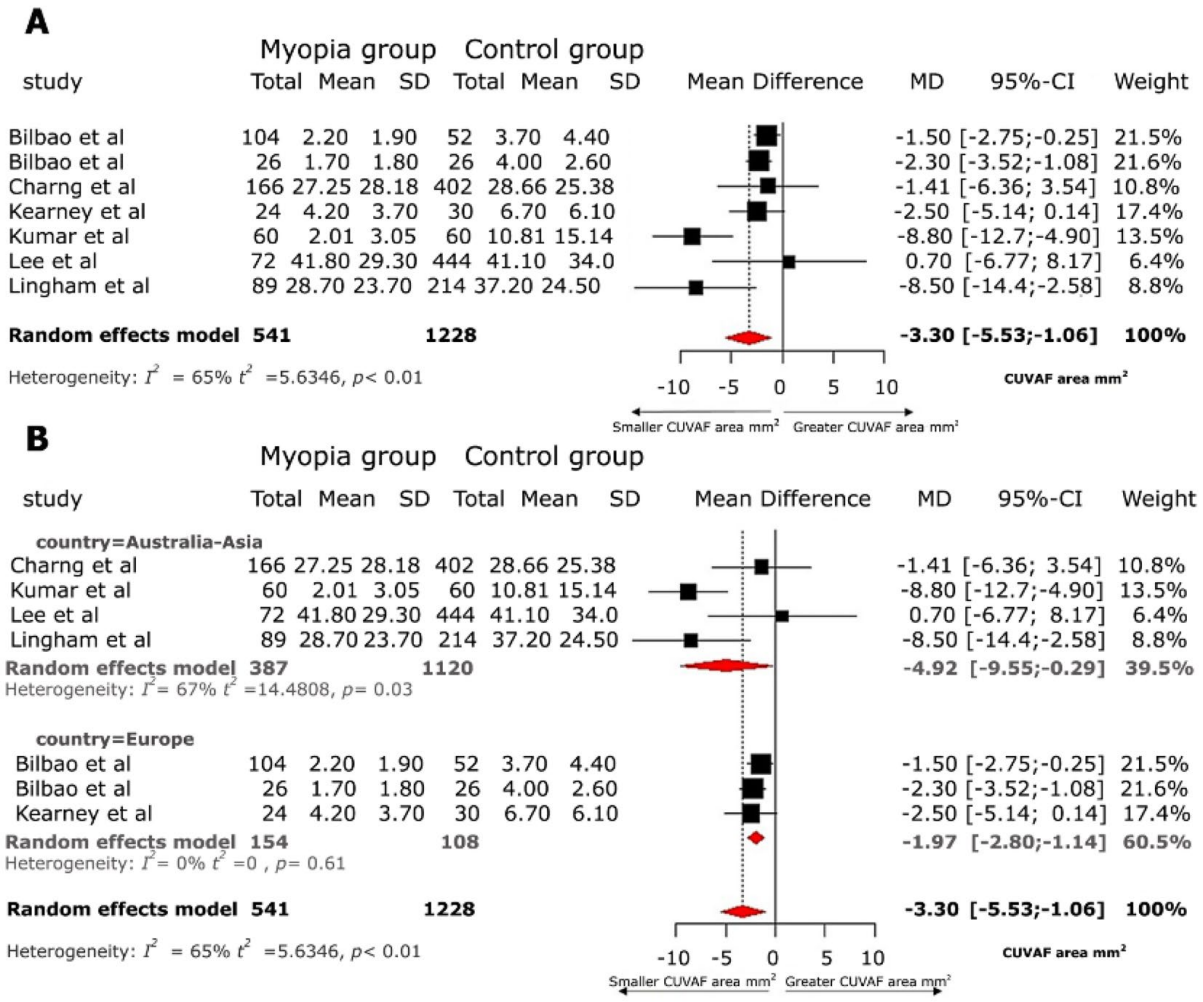
(**A**). Forest plot for the meta-analysis of the CUVAF area. Smaller CUVAF area (mm^2^) in the myopic group compared with the control group. (**B**) Forest plot for the meta-analysis of CUVAF area in patients with myopia compared with patients without myopia according to geographic location (Australia-Asia versus Europe). The myopic group has smaller CUVAF areas (mm^2^) than the control group.

The meta-analysis conducted found that myopic patients compared to non-myopic patients had statistically smaller CUVAF areas (mean difference of − 3.30 mm^2^, 95% CI − 5.53; − 1.06) (Fig. 3A). The mean CUVAF area in myopes from Australia and Asia was 24.73 mm^2^ (95% CI 8.35–41.12), while in emmetropes it was 29.47 mm2 (95% CI 16.33–42.61). This represents a relative difference of 16.08% less CUVAF area in myopes. In Europe, the mean CUVAF area of myopic patients was 2.55 mm^2^ (95% CI 1.23–3.86), and in emmetropes it was 4.55 mm^2^ (95% CI 2.98–6.12), representing in this case a relative difference of 43.95% less CUVAF area in myopes.

In the subgroup analysis, stratified by different geographic areas, it is evident that non-myopic and myopic patients from Australia and Asia, show a greater absolute difference in CUVAF area between them (mean difference of − 4.92 mm^2^ 95% CI − 9.55; − 0.29), compared to those from Europe (mean difference of − 1.97 mm^2^ 95% CI − 2.80; − 1.14) (Fig. 3B). This same analysis identified that the effect found in the studies carried out in Australia–Asia had greater statistical heterogeneity (I^2^ = 67%), compared to those carried out in Europe (Fig. 3B). In the meta-regression analysis, the difference in means of CUVAF areas that was identified presented a statistically significant difference (β 3.68; (p = 0.007) according to geographic regions (Supplementary Fig. 1).

When assessing the risk of publication bias using the funnel plot (Supplementary Fig. 2), an asymmetry in the size of the effects was evident, which could be explained by the statistical heterogeneity between the geographic location of the studies and the difference in the CUVAF areas between these regions.

**Figure 4:**
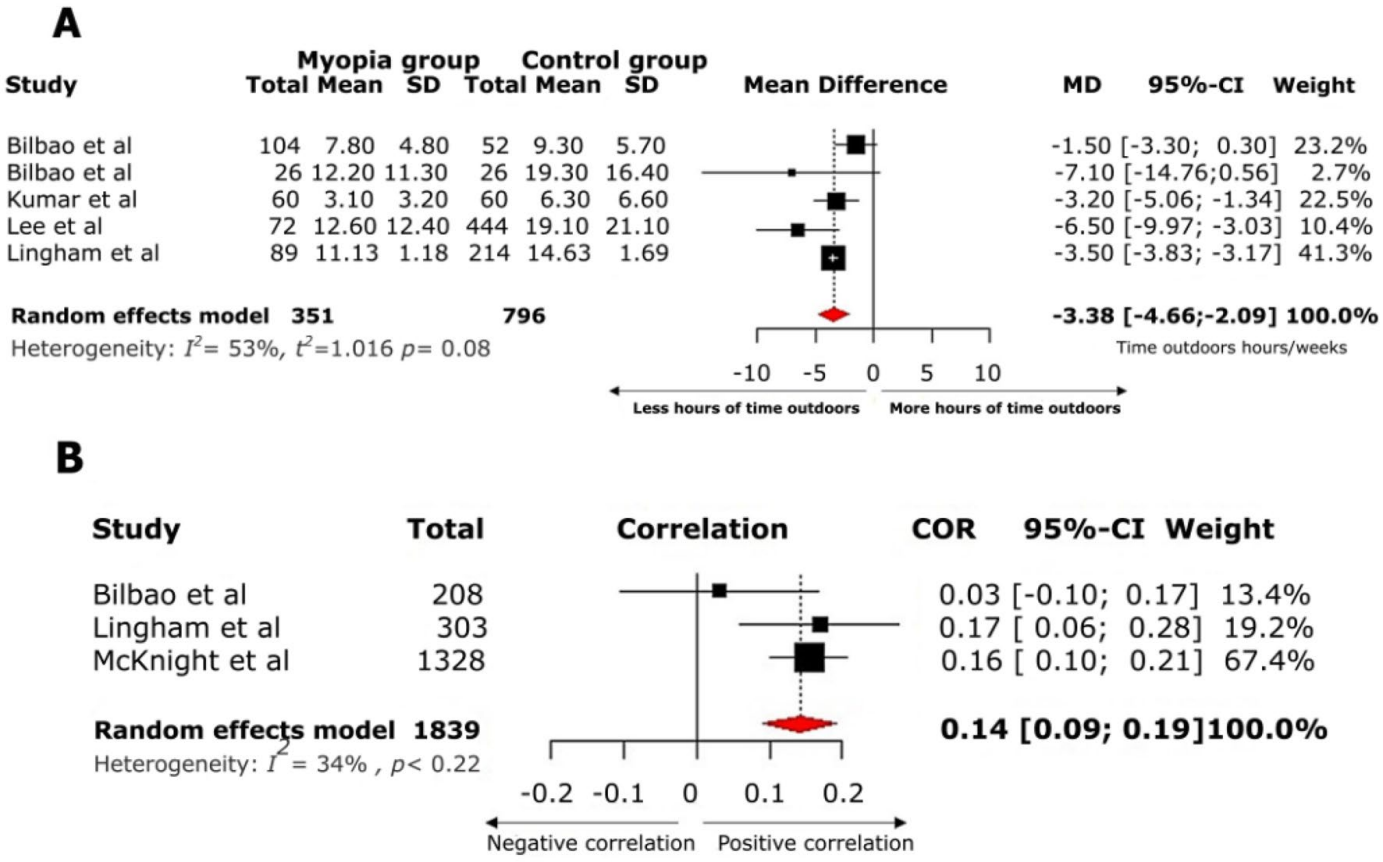
(**A**) Forest plot for the meta-analysis of time spent outdoors (h/week) in patients with myopia compared with patients without myopia. The myopic group reports less hours per week of outdoor exposure compared to the control group. (**B**) Forest plot of the correlation between CUVAF area (mm^2^) and time spent outdoors (h/week).

In the meta-analysis of outdoor exposure time, myopic patients reported less outdoor exposure time compared to non-myopic patients (mean difference − 3.38 h/week 95% CI − 4.66; 2.09) with a considerable statistical heterogeneity (I^2^ = 53%) (Fig. 4A). The correlation between CUVAF area and outdoor exposure time was analyzed in three articles, with a pooled coefficient of 0.14 (95% CI 0.09; 0.19) (Fig. 4 B).

### Risk of bias assessment

Risk of bias assessment. The studies of Bilbao et al.^31^ and Charng et al.^40^ were 100% compliant; while Mcknigth et al.^41^ Sherwin et al.^24^ and Yazar et al.^32^ had a compliance of 87.5% (with criteria for inclusion in the sample being the only item for which the studies did not comply). By means of the NOS star system, of the three cohort studies: one had a 9/9 star rating^33^ and two had 8/9 star rating^38,39^. The case–control study had a rating of 7/9 stars^34^.

## Discussion

The growing global concern over myopia demands investigation into modifiable factors influencing its development CUVAF^1,2,22,42,43^, a method for quantifying outdoor exposure, has been studied for myopia prevention, but varying cohort demographics in existing literature impede generalizable conclusions. This systematic review and meta-analysis examines the relationship between CUVAF area, myopia and time spent outdoors, while taking into account demographic factors affecting CUVAF size.

The evaluation of CUVAF as an objective biomarker for outdoor exposure time and its protective role in myopia prevalence yielded significant results in this meta-analysis. Non-myopic individuals showed 3.3 mm^2^ larger mean areas than myopic subjects, regardless of geographic solar exposure. A significant positive correlation between outdoor time, obtained from questionnaires, and CUVAF mean area was observed. Our comparative analysis confirmed the importance of the use of this type of method independently of the use of self-reported questionnaires.

The relationship between CUVAF and UV light exposure has been extensively investigated in myopia-related studies^34,44,45^, and other UV-induced ocular complications^24,46–48^. Moreover, the reliability and validity of CUVAF area measurement^23^ and its permanence for several months after exposure to sunlight have been demonstrated^27^. Violet light (360–400 nm wavelength), has been shown to prevent myopia progression in mice, chicks, and humans^49–52^. One meta-analysis shows that increased outdoor time is protective against the onset but not the progression of myopia, nevertheless, it is still unknown how much time outdoors is necessary to lower the incidence of myopia^49^.

A limitation of the use of CUVAF as a marker of myopia risk is the heterogeneity of CUVAF areas in different studies, which makes it difficult to create risk values applicable to the entire population. Nevertheless, our meta-analysis reveals that much of the variability can be attributed to different sun exposure duration, intensity, or wavelength reaching the eye in various world regions^53,54^. Sub-analysis by geographic area showed patients from Australia and Asia with higher UV radiation exposure had larger average CUVAF areas than Europeans. Previous studies have explored the effect of geographic location on CUVAF variability and have also observed that geographical areas closer to the equator, which receive a higher incidence of UV radiation, tend to present greater CUVAF areas, which contrast with the smaller CUVAF areas obtained in countries further away from the equator. These studies claimed that up to 50% of the variability observed in CUVAF area measurements between these regions are due to this geographical factor alone^55^. Moreover, not only is the mean CUVAF area different, but the variability between myopes and non-myopes is greater in regions with higher solar exposure, while in areas with low exposure, such as Europe, the measurements are more homogeneous. Creating region-specific normality tables would be a suitable solution to address this limitation.

However, it is important to clarify whether CUVAF is a measure of recent, subacute, or past exposure in order to apply this regional normality correction factor accurately in people who have lived in different regions throughout their lives. According to the literature, the natural history of CUVAF could be comparable to some ophthalmoheliosis lesions, where repair mechanisms may come into play reversing some of the UV-induced damage if the sun exposure is reduced over time. This could explain Charng and Sherwin results where the younger generation showed larger CUVAF areas. This finding likely suggests that CUVAF represents recent exposure to ocular UV radiation rather than lifetime cumulative exposure^45^.

Portable light meters have been used to measure outdoor time. Yet some studies have determined that a major drawback of these devices is that they cannot be used to accurately measure light exposure in large groups of people in real-world settings outside of clinical trials^56–58^. This is because the devices must be worn every day, which can be inconvenient and impractical. Some authors have suggested that measuring the blood levels of 25-hydroxyvitamin D3 can be used as an indicator of time spent outdoors, but this method is invasive, requires multiple blood samples, and its concentration in the body can be affected by other physiological factors independent of sunlight exposure^59,60^.

This review also addresses the question of whether smaller CUVAF areas in myopic subjects might be attributable to a protective effect from refractive correction including spectacles and contact lenses, or sunglasses. The researchers found that smaller CUVAF areas in myopic subjects were still observed when only participants who wore spectacles were included in the analysis^31^. Therefore, the use of refractive protection is not a confounding factor affecting CUVAF area; wearing glasses is not a reason for small CUVAF area. This suggests that perhaps conventional spectacles provide frontal but not adequate lateral protection against UV rays^31,41,61^. Even though UV-filtering contact lenses, may provide protection against this phenomenon, Lingham found that wearing contact lenses was not associated with changes in CUVAF area over time^61^, while Wolffsohn found no statistical difference between CUVAF in those wearing UV-blocking contact lenses compared to contact lenses with minimal UV blocking^28^. This leads us to believe that both direct UV incidence and the corneal focusing effect of peripheral light may contribute to the formation of CUVAF^62^. Furthermore, a study found that wearing sunglasses was associated with a faster decline in the CUVAF area, while other studies have not found this association^27,46,61,63^. The findings of the review suggest that conventional glasses and contact lenses may not provide adequate protection against UV radiation, and more research is needed to understand the protective effects of corrective lenses or sunglasses and CUVAF.

Interestingly, although all studies analyzed in this meta-analysis, with the exception of one, find a correlation between CUVAF area and SE, none of them were able to establish a correlation with AL, a parameter clearly related to the development of myopia^31,33,34^. We think that this result may be due to the greater variability of AL in the population, which would imply that larger sample sizes are needed to obtain statistically significant results. Another possible option could be that outdoor exposure is not only influencing AL, but also affecting the balance between AL and corneal curvature, as both are sources of refractive error. Therefore, the index that correlates these two could be a better measure to quantify biometric values and their correlation with CUVAF in order to extrapolate the impact on the SE parameter.

To our knowledge, this is the first systematic review and meta-analysis regarding the relationship between CUVAF, myopia and outdoor exposure. For the first time it is possible to statistically compare the differences in CUVAF area obtained in different continents with different degrees of sun exposure, demonstrating differences in both mean and variability of CUVAF area in different regions. Another advantage of the study is the completeness of our literature search. We believe that we included in the meta-analysis all observational studies between 2006 and 2022 which used the CUVAF technique as a biomarker of outdoor activity in myopes.

There are also some limitations in our study. As a result of only selecting patients without conjunctival alterations that could alter CUVAF measurement, our results cannot be extrapolated to patients with these pathologies. In addition, due to the lack of sufficient data in the included studies, we were unable to perform a subgroup analysis that differentiates CUVAF area between individuals with myopia and high myopia; however, some of the studies included in the review do perform this subanalysis and find statistically significant differences.

Our meta-analysis showed moderate heterogeneity, possibly reflective of the differences in the study designs, age, methods used, geographic location and ethnicities of the study populations. However, despite the high heterogeneity, a protective effect of outdoor time for myopia onset was established in the majority of studies. Some of the variability found in the studies is explained by the use of different devices for capturing images. The absence of a specific commercial device for this task leads to heterogeneous results. Moreover, it is also necessary to standardize the imaging processing algorithm used to quantify CUVAF area.

It is worth noting that the studies carried out so far have primarily explored the relationship between CUVAF and the prevalence of myopia but not its progression. It would be beneficial to conduct more longitudinal studies that provide deeper insights into the effect of CUVAF on the progression of myopia. Also, there is limited research of CUVAF in the child population, and myopia typically progresses during the first decades of life. It is important to conduct studies to better understand the relationship between solar exposure and myopia in children, and this information could be used to develop and implement effective preventive measures at the individual and public health level in schools.

This review highlights the usefulness of CUVAF as a biomarker from a statistical standpoint and also validates its use in future studies. In addition, a strong relationship was observed between CUVAF, myopia and time spent outdoors in different studies and geographic locations. Although different absolute CUVAF areas were observed (greater in Australia-Asia region) the correlation between higher CUVAF area and lower degree of myopia is observed in all the regions studied, validating CUVAF as a universal biomarker. The importance of this validation also extends to the fact that it is a simple, fast, and non-invasive quantitative method that could be useful to evaluate the compliance of outdoor activity programs as part of interventions to control the development and progression of myopia.

### Supplementary Information

Supplementary Information.

## Data Availability

The datasets used and/or analysed during the current study available from the corresponding author on reasonable request.

## Abbreviations

CUVAF: Conjunctival ultraviolet autofluorescence
UV: Ultraviolet
OCT: Optical coherence tomography
AL: Axial length
SE: Spherical equivalent
NOS: Newcastle Ottawa Scale

## References

[1] Holden BA (2016). Global prevalence of myopia and high myopia and temporal trends from 2000 through 2050. Ophthalmology.

[2] Ang, M. & Wong, T. Y. *Updates on Myopia A Clinical Perspective* (2020).

[3] Ang M (2020). Review: Myopia control strategies recommendations from the 2018 WHO/IAPB/BHVI Meeting on Myopia. Br. J. Ophthalmol..

[4] Smith TST, Frick KD, Holden BA, Naidoo KS (2009). Potential lost productivity resulting from the global burden of uncorrected refractive error. Bull. World Health Organ..

[5] Nemeth J (2021). Update and guidance on management of myopia. European Society of Ophthalmology in cooperation with International Myopia Institute. Eur. J. Ophthalmol..

[6] Zhang L (2020). Association between time spent outdoors and myopia among junior high school students. Medicine (Baltimore).

[7] Hua W-J, Jin J-X, Wu X-Y, Yang J-W, Jiang XGG-P (2015). Elevated light levels in schools have a protective effect on myopia. Ophthalm. Physiol. Opt..

[8] Williams KM (2015). Increasing prevalence of myopia in europe and the impact of education. Ophthalmology.

[9] Deng L, Pang Y (2019). Effect of outdoor activities in myopia control: Meta-analysis of clinical studies. Optom. Vis. Sci..

[10] Sherwin JC (2012). The association between time spent outdoors and myopia in children and a systematic review and meta-analysis. Ophtalmology.

[11] Rose KA, Morgan IG, Ip J, Kifley A (2008). Outdoor activity reduces the prevalence of myopia in children. Ophtalmology.

[12] Xiong S (2017). Time spent in outdoor activities in relation to myopia prevention and control: A meta-analysis and systematic review. Acta Ophthalmol..

[13] Karthikeyan SK, Ashwini DL, Priyanka M, Nayak A, Biswas S (2022). Physical activity, time spent outdoors, and near work in relation to myopia prevalence, incidence, and progression: An overview of systematic reviews and meta-analyses. Indian J. Ophthalmol..

[14] Wang W (2022). Myopia progression and associated factors of refractive status in children and adolescents in Tibet and Chongqing during the COVID-19 pandemic. Front. Public Health.

[15] Lanca C (2019). The effects of different outdoor environments, sunglasses and hats on light levels: Implications for myopia prevention. Trans. Vis. Sci. Tech..

[16] Biswas S (2023). A duration-dependent interaction between high-intensity light and unrestricted vision in the drive for myopia control. Investig. Ophthalmol. Vis. Sci..

[17] Zhou X, Pardue MT, Iuvone PM, Qu J (2017). Dopamine signaling and myopia development: What are the key challenges. Prog. Retin. Eye Res..

[18] Qian KW (2022). Altered retinal dopamine levels in a melatonin-proficient mouse model of form-deprivation myopia. Neurosci. Bull..

[19] Munteanu T (2018). Light-dependent pathways for dopaminergic amacrine cell development and function. Elife.

[20] Muralidharan AR (2021). Light and myopia: From epidemiological studies to neurobiological mechanisms. Ther. Adv. Ophthalmol..

[21] Fernández I (2022). Outdoor exposure in children from Buenos Aires Province, Argentina. Arch. Soc. Esp. Oftalmol..

[22] Demir P (2021). Refractive error, axial length, environmental and hereditary factors associated with myopia in Swedish children. Clin. Exp. Optom..

[23] Sherwin J (2012). Reliability and validity of conjunctival ultraviolet autofluorescence measurement. Br. J. Ophthalmol..

[24] Sherwin JC (2012). The association between time spent outdoors and myopia using a novel biomarker of outdoor light exposure. Clin. Epidemiol. Res..

[25] Lee S, Mackey D (2022). Prevalence and risk factors of myopia in young adults: Review of findings from the Raine study. Front. Public Health.

[26] Monici M (2005). Cell and tissue autofluorescence research and diagnostic application Cell and tissue autofluorescence research and diagnostic applications. Biotechnol. Annu. Rev..

[27] Haworth K, Chandler H (2017). Seasonal effect on ocular sun exposure and conjunctival UV autofluorescence. Optom. Vis. Sci..

[28] Wolffsohn JS, Drew T, Sulley A (2014). Conjunctival UV autofluorescence—prevalence and risk factors. Contact Lens Anterior Eye.

[29] Ju Lee O, Daya P, Shanel S, Mike O (2006). Ultraviolet fluorescence photography to detect early sun damage in the eyes of school-aged children. Ophtalmology.

[30] Lingham G (2019). Repurposing blue laser autofluorescence to measure ocular sun exposure. Clin. Exp. Ophthalmol..

[31] Bilbao V (2022). A cross-sectional observational study of the relationship between outdoor exposure and myopia in university students, measured by conjunctival ultraviolet autofluorescence (CUVAF ). J. Clin. Med..

[32] Yazar S (2014). Myopia is associated with lower vitamin D status in young adults. Invest. Ophthalmol. Vis. Sci..

[33] Kearney S (2018). Conjunctival ultraviolet autofluorescence area, but not intensity, is associated with myopia. Clin. Exp. Optom..

[34] Kumar S (2021). Myopia, melatonin and conjunctival ultraviolet autofluorescence: A comparative cross-sectional study in Indian myopes myopia, melatonin and conjunctival ultraviolet autofluorescence: a comparative. Curr. Eye Res..

[35] Stang A (2010). Critical evaluation of the Newcastle-Ottawa scale for the assessment of the quality of nonrandomized studies in meta-analyses. Eur. J. Epidemiol..

[36] Lo CKL, Mertz D, Loeb M (2014). Newcastle-Ottawa Scale: Comparing reviewers’ to authors’ assessments. BMC Med. Res. Methodol..

[37] Ma LL (2020). Methodological quality (risk of bias) assessment tools for primary and secondary medical studies: What are they and which is better?. Mil. Med. Res..

[38] Lingham G (2021). Time spent outdoors in childhood is associated with reduced risk of myopia as an adult. Sci. Rep..

[39] Lee SS-YY (2022). Incidence and progression of myopia in early adulthood. JAMA Ophthalmol..

[40] Charng J (2019). Estimation of heritability and familial correlation in myopia is not affected by past sun exposure. Ophthalm. Genet..

[41] Mcknight CM (2014). Myopia in Young adults is inversely related to an objective marker of ocular sun exposure: The Western Australian Raine cohort study. Am. J. Ophthalmol..

[42] Lingham G, Mackey DA, Lucas R, Yazar S (2020). How does spending time outdoors protect against myopia? A review. Br. J. Ophthalmol..

[43] Chen C (2022). Investigation on the prevalence and influencing factors of myopia among children and adolescents in Liyang city. Am. J. Transl. Res..

[44] Sun C (2017). Conjunctival ultraviolet autofluorescence as a measure of past sun exposure in children. Cancer Epidemiol. Biomark. Prev..

[45] Sherwin JC (2011). Distribution of conjunctival ultraviolet autoflourescence in a population-based study: The Norfolk Island Eye Study. Eye.

[46] McKnight CM (2015). Pterygium and conjunctival ultraviolet autofluorescence in young Australian adults: The Raine study. Clin. Exp. Ophthalmol..

[47] Stevenson LJ (2021). Has the sun protection Campaign in Australia reduced the need for pterygium surgery nationally?. Ophthalm. Epidemiol..

[48] Waszczykowska A, Bartosiewicz K, Podg M (2020). Conjunctival ultraviolet autofluorescence as a measure of riboflavin and ultraviolet and accelerated cross-linking exposure in keratoconic patients. J. Clin. Med..

[49] Cao K, Yue W, Yusufu M, Wang N (2019). Significance of outdoor time for myopia prevention: A systematic review and meta-analysis based on randomized controlled trials. Ophthalm. Res..

[50] Torii H, Ohnuma K, Kurihara T, Tsubota K (2017). Violet light transmission is related to myopia progression in adult high myopia. Sci. Rep..

[51] Strickland R (2020). Short-wavelength (violet) light protects mice from myopia through cone signaling. Invest. Ophthalmol. Vis. Sci..

[52] Jiang X (2021). Violet light suppresses lens-induced myopia via neuropsin ( OPN5) in mice. PNAS.

[53] Puthran SV, Biswas S, Karthikeyan SKTJ (2023). Association of sunlight exposure with visual impairment in an Indian fishing community. Indian J. Ophthalmol..

[54] Wong L (1993). Sunlight exposure, antioxidant status, and cataract in Hong Kong fishermen. J. Epidemiol. Community Health.

[55] Yazar S (2015). Genetic and environmental factors in conjunctival UV autofluorescence. JAMA Ophthalmol..

[56] Alvarez A, Wildsoet C (2014). Quantifying light exposure patterns in young adult students. J. Mod. Opt..

[57] Dharani R (2012). Comparison of measurements of time outdoors and light levels as risk factors for myopia in young Singapore children. Eye.

[58] Schmid KL (2013). Assessment of daily light and ultraviolet exposure in young adults. Optom. Vis. Sci..

[59] Tang SM (2018). Vitamin D and its pathway genes in myopia: Systematic review and meta-analysis. Br. J. Ophthalmol..

[60] Li X (2022). Low serum vitamin D is not correlated with myopia in Chinese children and adolescents. Front. Med.

[61] Lingham G (2021). Conjunctival ultraviolet autofluorescence area decreases with age and sunglasses use. Br. J. Ophthalmol..

[62] Sliney DH (2002). How light reaches the eye and its components. Int. J. Toxicol..

[63] Kearney S (2016). The use of conjunctival ultraviolet autofluorescence (CUVAF) as a biomarker of time spent outdoors. Ophthalm. Physiol. Opt..

