# Supplementary material for: Conjunctival ultraviolet autofluorescence as a biomarker of outdoor exposure in myopia: a systematic review and meta-analysis
Source: Sci Rep. 2024 Jan 11;14:1097. doi: 10.1038/s41598-024-51417-9 (PMC10784576; doi:10.1038/s41598-024-51417-9)
Supplement: Supplementary file 1 — Supplementary Information. [file 41598_2024_51417_MOESM1_ESM.pdf]

SUPPLEMENTAL CONTENT

Supplementary Figure 1

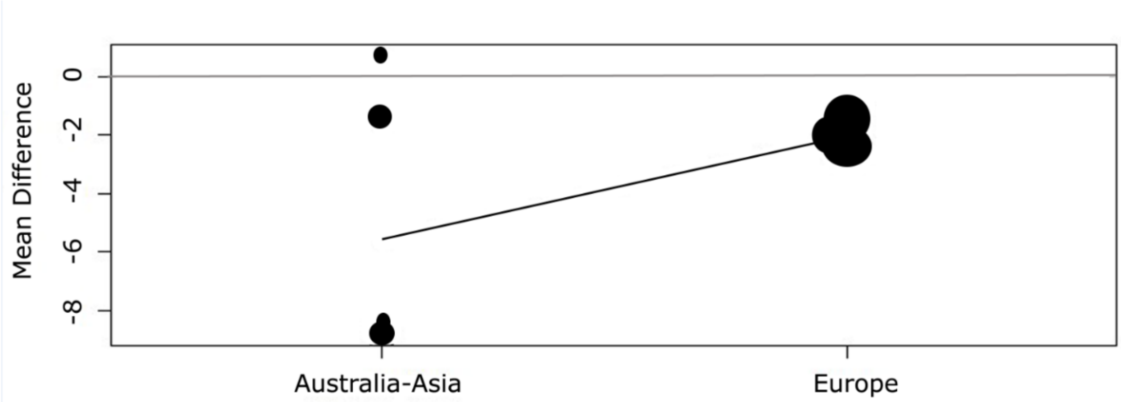

Supplementary Figure 1. Regression line of the difference in mean CUVAF area by Australia-Asia and Europe regions.

Supplementary Figure 2

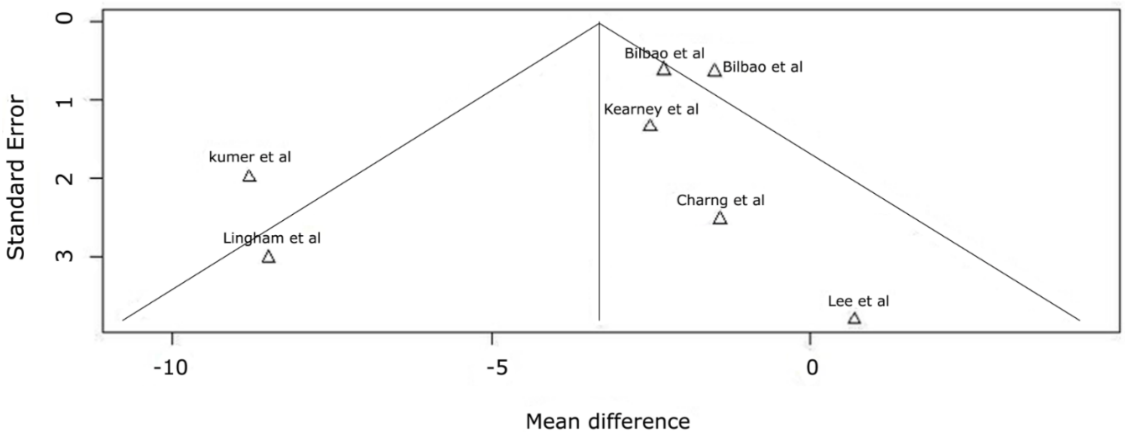

Supplementary Figure 2. Funnel plot for the included studies evaluating the mean difference of CUVAF area between myopes and non-myopes, showing an asymmetry in effect size.
